# Identification and Validation of Reference Genes for Reliable RT-qPCR Normalization in *Schisandra chinensis* Across Different Tissues and Abiotic Stress Conditions

**DOI:** 10.3390/plants15131946

**Published:** 2026-06-24

**Authors:** Longjun Liang, Xin Song, Xuanhe Zhang, Yingchun Liu, Guangli Shi, Zhenxing Wang, Cong Zhang, Chengzhan Li, Xiyu Zhang, Dan Sun, Jun Ai

**Affiliations:** College of Horticulture, Jilin Agricultural University, Changchun 130118, China15943017532@163.com (X.S.); 18241231763@163.com (X.Z.); 17649830071@163.com (Y.L.); shiguanglii@163.com (G.S.); zhenxingw@jlau.edu.cn (Z.W.); 15344387133@163.com (C.Z.); 15567386239@163.com (C.L.); 18561106781@163.com (X.Z.)

**Keywords:** normalization, housekeeping genes, expression stability, gene validation, transcriptional analysis

## Abstract

Reverse transcription quantitative real-time PCR (RT-qPCR) is a highly efficient and sensitive technique for quantifying gene transcript levels. The accuracy of gene expression analysis depends critically on the selection of appropriate reference genes for normalization, which is essential to minimize technical variation arising from differences in RNA quality, reverse transcription efficiency, and sample handling. *Schisandra chinensis* is a medicinally important plant with a long history of use in traditional Chinese medicine and has gained increasing global recognition. In recent years, a growing number of studies have employed molecular biology approaches to investigate the molecular mechanisms underlying secondary metabolite biosynthesis in *S. chinensis*. However, systematically validated reference genes for RT-qPCR analysis in this species have not yet been established. In the present study, the expression stability of eleven candidate reference genes was evaluated across different tissues and under various abiotic stress conditions in *S. chinensis* using four statistical algorithms: geNorm, NormFinder, BestKeeper, and RefFinder. Comprehensive analysis revealed that *PP2A15* and *UBC2* were the optimal reference gene combination for leaves; *UBC2* and *UBC11* for stems; *RPL6* and *PP2A15* for roots; *RPL21* and *RPL6* for fruits; and *RPL6* and *UBC11* as the best-performing pair across all tissue types. Under abiotic stress conditions, *UBC11* and *UBC2* exhibited the highest stability in both leaves and roots under salt stress; *UBC2* and *GPN1* proved most stable under alkaline stress; *UBC2* and *RPL6* were identified as the most suitable combination under drought stress; and *UBC2* and *UBQ12* demonstrated consistently stable expression across all three abiotic stress treatments. The reliability of these reference gene combinations was further validated by examining the expression profiles of three target genes. Collectively, these findings establish a validated reference gene toolkit for future gene expression studies in *S. chinensis*, particularly for the functional characterization of genes involved in lignan biosynthesis and abiotic stress responses.

## 1. Introduction

*Schisandra chinensis* (Turcz.) Baill., a valuable plant resource, is found in Northern China, Korea, Japan, Eastern parts of Russia, and North Korea. This perennial deciduous woody vine, commonly known as “Wuweizi” in China, holds significant economic and medicinal importance because of its prominent presence in the market. Since ancient times, the herb has been used for its beneficial chemical components, such as lignans, volatile oils, polysaccharides, and organic acids [1]. Additionally, polysaccharides from *S. chinensis* could enhance immunoglobulin and cytokine levels, induce cytokine production in RAW264.7 cells in vitro, and activate anti-tumor activity [2]. Schisandrin A could synergistically enhance the effects of the drug gefitinib, inducing apoptosis in lung cancer cells and inhibiting the IKKB/NF-KB signaling pathway [3]. These extensive studies highlight the significant medicinal properties of *S. chinensis*, which helps to further explore its value in both traditional and modern medicine.

Real-time quantitative PCR (RT-qPCR) is a fluorescence-based technique that enables real-time monitoring of amplicon accumulation throughout the amplification process, allowing relative transcript quantification via standard curve construction. Its primary advantages include high sensitivity, excellent repeatability, the capability to analyze a large number of samples, and a higher detection rate compared to conventional methods. This makes it particularly useful for analyzing gene expression levels. However, multiple factors can compromise the accuracy of RT-qPCR quantification, including sample quality and concentration, reverse transcription efficiency, amplification protocol, and experimental settings. To control for these variables and normalize target gene expression, it is essential to identify and validate stably expressed reference genes. Accordingly, RT-qPCR analysis incorporating appropriate reference genes is critical for accurate transcript quantification [4]. Reference genes have traditionally been selected from so-called housekeeping genes involved in fundamental cellular processes. Yet, as demonstrated by Long et al. [5], Wang et al. [6], and Chen et al. [7], the expression stability of these reference genes varies considerably across different species, tissue types, developmental stages, and experimental treatments. This pronounced variability underscores the necessity of systematically selecting and validating reference genes tailored to specific experimental contexts, thereby ensuring accuracy and reliability in RT-qPCR-based gene expression analysis.

In recent years, increasing attention has been paid to the molecular biology of *S. chinensis*. For instance, Li et al. [8] sequenced the transcriptome of *S. chinensis* fruits at different developmental stages and established a comprehensive transcriptome database, identifying 26 key enzyme gene sequences that were differentially expressed in the lignin biosynthesis pathway. Subsequently, Shi et al. [9,10] constructed an EST-SSR marker library and investigated the relationship between lignan content and the expression of genes involved in lignan biosynthesis in *S. chinensis*. Furthermore, Cheng et al. [11] integrated metabolomics, transcriptomics, and DNA molecular marker technologies to explore sexual dimorphism in *S. chinensis* and developed DNA molecular markers for sex identification. Despite these advances, suitable reference genes for RT-qPCR normalization in *S. chinensis* have not yet been systematically identified.

To address this gap, the present study evaluated the expression stability of eleven candidate reference genes across different tissue samples and under various abiotic stress conditions. These candidate genes, including glyceraldehyde-3-phosphate dehydrogenase 1 (*GPN1*), protein phosphatase 2A15 (*PP2A15*), 60S ribosomal protein L6 (*RPL6*), 60S ribosomal protein L15 (*RPL15*), 60S ribosomal protein L21 (*RPL21*), tubulin alpha-2 (*TUBBA2*), tubulin beta-4 (*TUBB4*), ubiquitin-conjugating enzyme 2 (*UBC2*), ubiquitin-conjugating enzyme 11 (*UBC11*), ubiquitin 12 (*UBQ12*), and actin (*AcTIN*), were selected based on two complementary criteria. First, all candidates were identified from the RNA-Seq transcriptome profiling of *S. chinensis* (unpublished data), with priority given to genes showing relatively stable expression across different tissues and stress conditions. Second, each gene belongs to a functional category with documented precedence as a stable reference gene in related plant species. *GPN1*, *TUBBA2*, *TUBB4*, and *AcTIN* represent classic housekeeping genes widely used for RT-qPCR normalization, encoding glyceraldehyde-3-phosphate dehydrogenase, α/β-tubulin, and actin, respectively [12,13,14]. *PP2A15* was included because of its proven stability under diverse abiotic stresses in multiple plant species, including *Populus euphratica* [15], creeping bentgrass [16], pearl millet [17], and *Momordica charantia* [18]. *UBQ12* was selected based on the consistent expression of ubiquitin-family genes in woody and vine species such as grape [19,20] and apple [21]. In addition, the ubiquitin-conjugating enzyme genes *UBC2* and *UBC11*, together with the 60S ribosomal protein genes *RPL6*, *RPL15*, and *RPL21*, were chosen because ribosome and ubiquitination related genes have recently emerged as superior alternatives to traditional reference genes in wheat [5], kiwifruit [7], okra [22], and cotton [23]. Thus, this dual strategy, combining transcriptome guided selection with literature-supported functional relevance, ensured a broadly representative panel of candidate reference genes for stability evaluation in *S. chinensis*. The expression stability of these candidates was then assessed using four commonly used algorithms: BestKeeper, geNorm, NormFinder, and RefFinder. This approach is essential for identifying stable and suitable reference genes for RT-qPCR data normalization, thereby providing a reliable foundation for more accurate gene expression analyses in future studies of *S. chinensis*.

## 2. Materials and Procedures

### 2.1. Plant Materials

Four-year-old cuttings of the *S. chinensis* variety ‘Zaohong’ were collected from the *S. chinensis* germplasm database at Jilin Agricultural University, which is situated in Changchun City, Jilin, China (coordinates 43°48′005″ N, 125°24′15″ E). Fifty days after flowering (DAF), samples of leaves, stems, roots, and fruitlets (FS1) were gathered. Furthermore, at 80 DAF and 110 DAF, respectively, fruits at the color transition stage (FS2) and ripe fruits (FS3) were gathered (Supplementary Figure S1). Three biological replicates of each sample were used to guarantee the data’s integrity. Following collection, the samples were immediately cleaned, submerged in liquid nitrogen, frozen, and stored in a freezer at −80 °C for preservation and later analysis. The careful technique of gathering and preserving the samples was essential to preserving their biological integrity and guaranteeing accurate results.

### 2.2. Abiotic Stress Treatments

The stress treatments were applied to well-established potted plants that had been growing under standard greenhouse conditions. Two-year-old cuttings of the ‘Zaohong’ variety were chosen as test materials for the investigation into the abiotic stress responses. The cuttings were treated with indole-3-butyric acid (IBA) at 1000 mg/L (quick-dip method) to promote rooting prior to transplanting. The two-year-old cuttings had fully developed root systems and were in active vegetative growth at the time of treatment. Three different single-factor abiotic stress treatments were applied to these cuttings: drought stress, alkaline stress, and salt stress. The concentrations of 200 mmol/L NaCl and 200 mmol/L NaHCO_3_ were selected based on preliminary dose–response experiments in which *S. chinensis* cuttings were exposed to a concentration gradient (50, 100, 150, 200, 300 mmol/L) of each stressor. At 200 mmol/L, plants exhibited clear stress symptoms (leaf chlorosis, reduced growth rate) within the 15-day treatment period without reaching mortality, making it suitable for evaluating sub-lethal stress responses. For every treatment, the particular steps were as follows:

(1) Salt stress treatment: the cuttings were irrigated with a 200 mmol/L NaCl solution. This concentration was chosen to simulate salt stress conditions.
(2) Alkaline stress treatment: the cuttings were watered with a 200 mmol/L NaHCO_3_ solution, with the pH maintained at approximately 7.8, to simulate alkaline stress.
(3) Drought stress treatment: drought stress was imposed by completely withholding irrigation for 15 days, during which the soil relative water content (SRWC) was monitored gravimetrically. The SRWC declined from approximately 80% (field capacity) at day 0 to approximately 25% at day 12, representing a progression from mild to moderate-severe drought stress.

The control group consisted of cuts that were not under any kind of stress. Every three days, the soil in the cultivation bowls was completely saturated with the relevant solutions for each stress treatment. This irrigation schedule was the same for every treatment. For each stress treatment, three biological duplicates were carried out to guarantee thorough data collection. The roots and leaves of the cuttings were gathered at specific intervals: 0, 3, 6, 9, and 12 days following the initiation of the stress treatment. The test items were gathered, cleaned, and air-dried before being swiftly frozen in liquid nitrogen. They were then maintained at −80 °C in a refrigeration unit. The careful collecting, processing, and storage of the materials was essential to preserving their integrity.

### 2.3. RNA Extraction and cDNA Synthesis

The RNA extraction process from *S. chinensis* involved the utilization of the RNA prep Pure Polysaccharide Polyphenol Plant Total RNA Extraction Kit, a product from Tiangen Biochemical Co., Ltd. (Beijing, China). A 2% agarose gel electrophoresis analysis was conducted for evaluating RNA integrity. Subsequently, the high-quality total RNA was utilized for the cDNA synthesis process. Transgen Biotech Co., Ltd. (Beijing, China) High-Temperature Resistant Full Pre-mixed First Strand cDNA Synthesis Kit was used in this phase. All the cDNA samples were then stored at a temperature of −80 °C, which is the optimal condition for long-term preservation. These rigorous procedures were essential to guarantee the accuracy and reliability of the subsequent Retrotranscribed Quantitative Polymerase Chain Reaction (RT-qPCR) assessment in the research on *S. chinensis*.

### 2.4. Primer Design for Reference Genes

Eleven housekeeping genes were chosen as potential reference genes according to the existing literature on reference genes and the transcriptome sequencing data of *S. chinensis*. These candidate reference genes include *GPN1*, *PP2A15*, *RPL6*, *RPL15*, *RPL21*, *TUBBA2*, *TUBB4*, *UBC2*, *UBC11*, *UBQ12*, and *AcTIN*. Primer3 and Primer5 tools were utilized to design the RT-qPCR primers following established principles (Table 1). To prepare the RT-qPCR template, the cDNA from all samples were evenly mixed. The generated template was then diluted five times for each gradient, resulting in five distinct gradients. Subsequently, RT-qPCR reactions were conducted and Ct values obtained. These values were employed to construct a reference curve, enabling us to calculate the amplification efficiency (*E*) and correlation coefficient (*R*^2^) of the designed primers. Simultaneously, the specificity of the PCR products generated by the primers were verified through 2% agarose gel electrophoresis, which ensured the accurate amplification of the intended target sequences by the primers. These rigorous procedures were essential for verifying the suitability and reliability of the selected reference genes and primers for the subsequent analysis of gene expression in the study of *S. chinensis*.

### 2.5. RT-qPCR Assay

In the RT-qPCR tests to assess the reference genes, the Fluorescence Quantitative Premix Reagent Kit from Transgen Biotech Co., Ltd. (Beijing, China) was employed. The quantitative procedures were carried out employing the Applied Biosystems^®^ StepOnePlus Real-Time PCR System, a reliable platform for real-time fluorescence quantitative PCR. For each sample, three replicates were conducted to ensure robust data consistency. Additionally, No-Template Controls (NTC) were included as controls in each sample to monitor for potential contamination. The reaction system for each RT-qPCR assay consisted of a cumulative volume of 20 µL, which included the following components: 10 µL of 2 × PerfectStart Green qPCR Super Mix, 1 µL of cDNA, 0.4 µL of Passive Reference Dye (50 ×) (optional), 0.4 µL of each primer, and 9.4 µL of Nuclease-Free Water. This meticulously composed reaction system ensured accurate and reproducible results. The RT-qPCR program was executed according to the following parameters: a first denaturation step for a duration of 30 s at 94 °C, sequentially 40 cycles of denaturation at 94 °C for 5 s and annealing/extension for a duration of 30 s at 60 °C. Additionally, a dissociation curve was produced by progressively elevating the temperature from 65 °C to 95 °C. Each RT-qPCR assessment was conducted three times to ensure the reliability of the obtained data.

### 2.6. Validation of Candidate Reference Genes

To confirm the credibility of the suggested reference genes, RT-qPCR analysis was conducted to evaluate the expression profiles of three target genes: Pineolin lignin reductase 1 (*PLR1*), Dirigent 3 (*DIR3*), and Mitogen-activated protein kinases 4 (*MPK4*). *PLR1* and *DIR3* were chosen due to their upstream involvement in lignan biosynthesis, which is a crucial process in lignan production [24,25,26]. *MPK4* was derived from the *S. chinensis* transcriptome (unpublished) as it plays a crucial role in plant responses to abiotic stress [27]. According to the RefFinder analysis, *RPL6* and *UBC11* have been identified as the most reliable reference genes in different organs, and the least stable reference gene was *TUBB4*. The highest-ranked reference genes independently, as well as a composite selection of the four most stable reference genes (*UBC2*, *UBQ12*, *PP2A15*, and *UBC11*), along with the least stable reference gene (*TUBB4*), were investigated under abiotic stress. The relative expression levels were computed with the 2^−ΔΔCt^ method, based on data obtained from three biological and technical duplicates [28].

### 2.7. Statistical Analyses

All RT-qPCR experiments were performed with three biological replicates, each assayed in three technical replicates. The raw quantification cycle (Ct) values were initially examined using box plots to visualize the expression distribution of the eleven candidate reference genes across all sample groups. All data are presented as mean ± standard deviation (SD). Statistical significance of differences in relative gene expression was assessed using [one-way ANOVA followed by Tukey’s HSD test/Student’s *t*-test] with a significance threshold of *p* < 0.05. The expression stability of candidate reference genes was comprehensively evaluated using four independent statistical algorithms.

geNorm analysis: The geNorm algorithm [29] calculates the gene expression stability value (M) based on the average pairwise variation between a given gene and all other candidate reference genes. Lower M values indicate higher expression stability, and genes with M values below the threshold of 1.5 are considered suitable as reference genes. To determine the optimal number of reference genes required for reliable normalization, pairwise variation analysis (V*n*/V*n* + 1) was performed, with a cutoff value of 0.15, below which the inclusion of an additional reference gene does not significantly improve normalization accuracy.

NormFinder analysis: The NormFinder algorithm [30] employs a model-based approach to estimate both intra-group and inter-group expression variation. Candidate reference genes are ranked according to their stability values, with lower values representing more stable expression across the tested experimental conditions. Unlike geNorm, NormFinder accounts for systematic differences among sample subgroups, thereby reducing the risk of selecting co-regulated genes as the best reference pair.

BestKeeper analysis: The BestKeeper algorithm [31] evaluates expression stability based on the standard deviation (SD) and coefficient of variance (CV) of raw Ct values. Candidate reference genes with SD values below 1.0 are regarded as stably expressed across the tested conditions. In addition, BestKeeper performs Pearson pairwise correlation analysis between each candidate gene and the BestKeeper Index, which is calculated as the geometric mean of Ct values of the most stable genes.

RefFinder comprehensive ranking: RefFinder (http://blooge.cn/RefFinder/) (accessed on 10 October 2025) is a web-based tool that integrates the outputs from geNorm, NormFinder, and BestKeeper to provide a comprehensive stability ranking [32]. It assigns each gene an appropriate weight based on its ranking from the individual algorithms and calculates the geometric mean of these ranks, thereby generating a consensus ranking that is more robust than any single-algorithm evaluation.

## 3. Results

### 3.1. Evaluation of Reference Gene Amplification Efficiency and Primer Specificity

**Figure 1:**
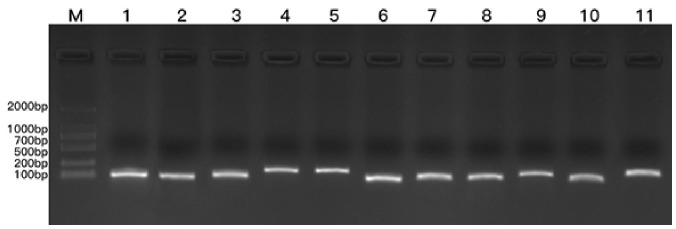
Agarose gel electrophoresis detection for the PCR products of eleven candidate reference primers in *S. chinensis* (M: DL2000 marker, 1: *GPN1*, 2: *PP2A15*, 3: *RPL6*, 4: *RPL15*, 5: *RPL21*, 6: *TUBBA2*, 7: *TUBB4*, 8: *UBC2*, 9: *UBC11*, 10: *UBQ12*, 11: *AcTIN*).

**Figure 2:**
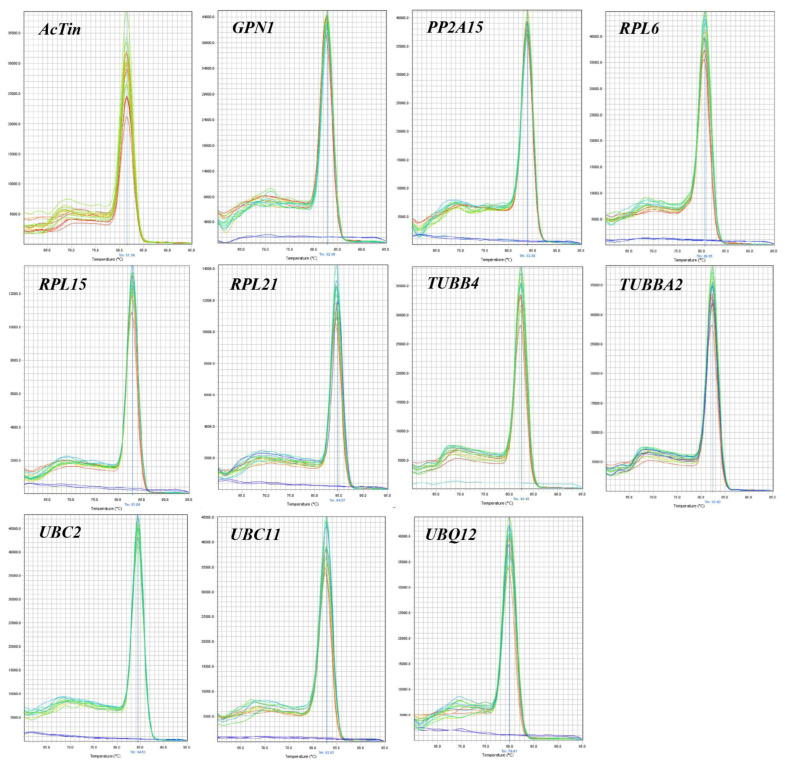
Melting curves of eleven candidate reference genes. Different colored lines correspond to individual biological replicates; single sharp peaks confirm specific amplification of each target gene.

To verify the specificity of each candidate reference gene, conventional RT-PCR was first performed, followed by resolution of the amplification products on 2% agarose gel electrophoresis. A primer pair was deemed suitable for downstream assays only when a single, well-defined band was observed (Figure 1). Consistent with this, melt-curve analysis revealed that all 11 target genes produced a single sharp peak, with no evidence of primer-dimer formation or non-specific amplification (Figure 2). The correlation coefficient (*R*^2^) ranged from 0.992 to 0.999, and the amplification efficiency (*E*%) varied from 98.6% (*TUBBA2*) to 105.4% (*RPL15*). As summarized in Table 1 and Supplementary Figure S2, these parameters fell within the accepted ranges (90% < *E*% < 110%; *R*^2^ > 0.99).

### 3.2. Profile of Reference Genes Expression

**Figure 3:**
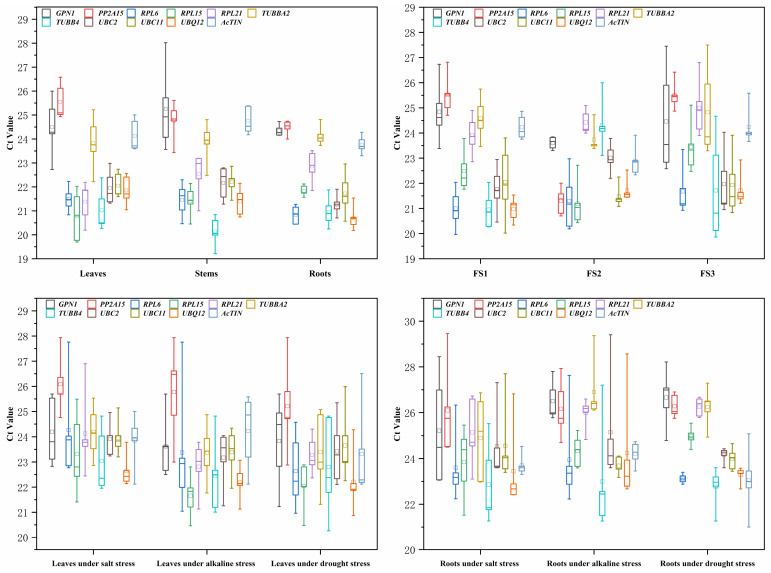
Ct value distribution for eleven candidate reference genes. The central line within the box corresponds to the median Ct value. The smaller rectangles symbolize the mean value. The top and bottom boundaries of the box denote the 25th and 75th percentiles, while the whiskers illustrate the highest and lowest values, respectively.

The Ct (quantification cycle) value represents the cycle number at which the fluorescence signal crosses the detection threshold during PCR amplification; lower Ct values reflect higher transcript abundance of the target gene. As shown in Figure 3, the mean Ct values of the eleven candidate reference genes ranged from 22.19 to 25.22 across all samples, with coefficients of variation (CV) spanning 4.3% to 7.8%. Tissue-specific analysis revealed the following mean Ct ranges: 20.77 (*RPL15*) to 25.54 (*PP2A15*) in leaves, 20.14 (*TUBB4*) to 25.25 (*GPN1*) in stems, 20.71 (*UBQ12*) to 24.48 (*PP2A15*) in roots, 19.96 (*RPL6*) to 26.81 (*PP2A15*) in FS1, 20.19 (*RPL6*) to 25.99 (*TUBB4*) in FS2, and 20.83 (*UBC11*) to 27.49 (*TUBBA2*) in FS3. Notably, these candidate genes exhibited not only tissue-dependent expression divergence but also pronounced instability under abiotic stress treatments. Under stress conditions, mean Ct values ranged from 22.64 (*UBQ12*) to 26.08 (*PP2A15*) in salt-stressed leaves, 22.86 (*TUBB4*) to 26.09 (*PP2A15*) in salt-stressed roots, 21.65 (*RPL15*) to 25.77 (*PP2A15*) in alkaline-stressed leaves, 22.99 (*TUBB4*) to 26.90 (*TUBBA2*) in alkaline-stressed roots, 22.03 (*RPL15*) to 25.21 (*PP2A15*) in drought-stressed leaves, and 22.75 (*TUBB4*) to 26.65 (*GPN1*) in drought-stressed roots.

### 3.3. Expression Stability of Candidate Reference Genes

#### 3.3.1. GeNorm Analysis

**Figure 4:**
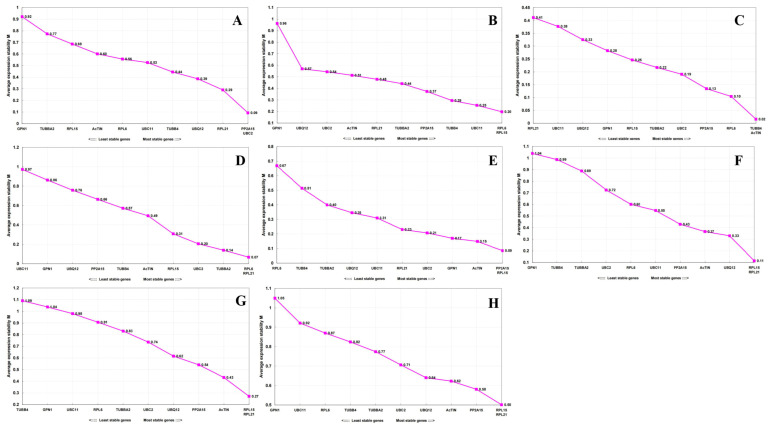
The mean expression stability values (M) for the eleven candidate reference genes were calculated by geNorm in various tissues. (**A**): Leaves; (**B**): Stems; (**C**): Roots; (**D**): FS1; (**E**): FS2; (**F**): FS3; (**G**): Fruits; (**H**): All samples. The horizontal arrow under each panel shows the stability order: left = least stable gene (higher *M*), right = most stable gene (lower *M*); smaller *M* value indicates superior expression stability.

**Figure 5:**
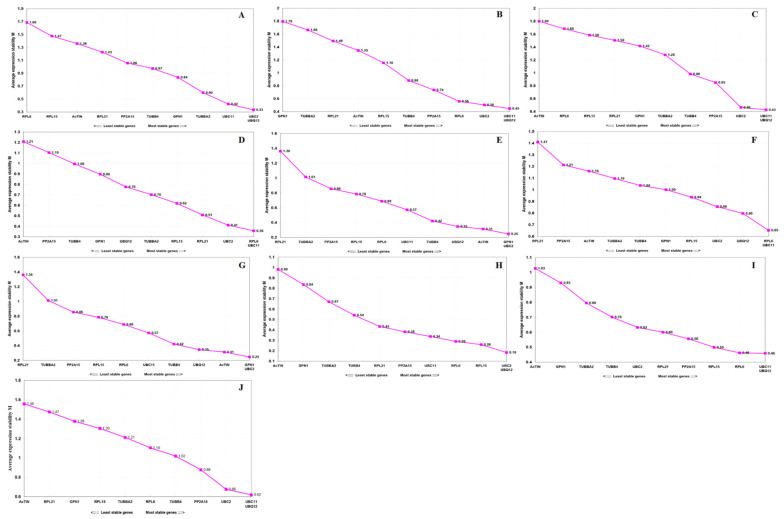
The mean expression stability values (M) for the eleven candidate reference genes were calculated by geNorm in leaves and roots under various abiotic stress conditions. (**A**): Leaves under salt stress; (**B**): Roots under salt stress; (**C**): Leaves and roots under salt stress; (**D**): Leaves under alkaline stress; (**E**): Roots under alkaline stress; (**F**): Leaves and roots under alkaline stress; (**G**): Leaves under drought stress; (**H**): Roots under drought stress; (**I**): Leaves and roots under drought stress. (**J**): Leaves and roots under various abiotic stress sets. The horizontal arrow under each panel shows the stability order: left = least stable gene (higher *M*), right = most stable gene (lower *M*); smaller *M* value indicates superior expression stability.

The GeNorm software (https://genorm.cmgg.be/) (accessed on 10 October 2025) was employed to calculate the expression stability of each genes by value (M). The M value exhibits an inverse relationship with the expression stability of the reference genes, with smaller M values indicating higher stability of the reference gene. A potential reference gene is considered suitable as a candidate reference gene if the M value is less than 1.5, and its expression stability is then examined [29]. The M values for eleven reference genes across various samples and abiotic stress conditions are presented in Figure 4 and Figure 5. It is evident that the most stable genes are *PP2A15* and *UBC2* (M = 0.09), whereas the most unstable gene in leaves is *GPN1* (M = 0.92). *GPN1* (M = 0.96) stands out as the least stable gene in stems, whereas *RPL6* and *RPL15* (M = 0.20) are shown to be the most reliable genes. *TUBB4* and *AcTIN* (M = 0.02) show remarkable stability in roots, whereas *RPL21* (M = 0.41) is the least stable gene. In the context of integrated fruit events, *RPL15* and *RPL21* (M = 0.27) are recognized as the most stable reference genes, while *TUBB4* (M = 1.09) is distinguished as the least stable gene due to its significant instability. *RPL15* and *RPL21* (M = 0.50) are the most stable genes among the various organ sets, while *GPN1* (M = 1.05) has the unfortunate distinction of being the least stable gene among these several tissues. Under salt stress, the most stable genes in leaves are *UBC2* and *UBQ12* (M = 0.33), while the least stable gene is *RPL6* (M = 1.69). On the other hand, *GPN1* (M = 1.79) exhibits the lowest level of root stability, while *UBC11* and *UBQ12* (M = 0.45) demonstrate the highest level of stability. *RPL6* and *UBC11* had the lowest M values in leaves (0.36) under alkaline stress, while *AcTIN* had the lowest M values (1.21). *GPN1* and *UBC2* (M = 0.25) exhibit the highest levels of stability in root, while *RPL21* (M = 1.36) is the least stable. *GPN1* and *UBC2* (M = 0.25) are identified as the most stable genes in leaves under drought stress, whereas *RPL21* (M = 1.36) shows the least stability. On the other hand, *AcTIN* (M = 0.98) exhibits the least stability in roots, while *UBC2* and *UBQ12* (M = 0.18) rank highest in stability. While *AcTIN* continues to be the most unstable gene in both leaves and roots, *UBC11* and *UBQ12* consistently exhibit the highest levels of stability under these three abiotic stress conditions.

#### 3.3.2. NormFinder, BestKeeper and Comprehensive Analysis of Candidate Reference Genes

NormFinder examines and ranks the stability of potential reference genes based on their expression levels. It achieves this by assessing inter- and intra-group variations [30]. In Table 2, the most reliable genes in various plant parts could be observed as follows: *PP2A15* and *UBC2* in leaves (consistent with geNorm results), *UBC2* and *UBQ12* in stems, *RPL6* and *PP2A15* in roots, *RPL21* and *TUBBA2* in fruits (Supplementary Table S1), and *UBC11* and *RPL6* in different tissues. Whereas *GPN1* exhibits the least stability in leaves, *RPL21* in stems, *PP2A15* in roots, *TUBB4* in fruits, and *GPN1* in different tissues. In the context of abiotic stress conditions, *TUBBA2* and *UBC11* are most reliable in leaves, while *RPL6* and *UBC2* excel in roots under salt stress. Under alkaline stress, *TUBBA2* and *RPL21* are the most stable genes in leaves, while *TUBBA2* and *GPN1* rank first in roots. For drought stress, *RPL15* and *RPL6* are stable in leaves, while *UBC2* and *UBQ12* are the two best reference genes in roots (consistent with geNorm findings) (Supplementary Tables S2–S4). Furthermore, for these three stress treatment sets, *UBC2* and *UBQ12* were identified as the stable genes in both leaves and roots, which is consistent with geNorm (Table 3 and Figure 5). BestKeeper evaluates the stability of each potential reference gene’s expression by calculating the SD and CV of Ct values associated with these candidate reference genes across diverse experimental treatments. When subjected to scrutiny via BestKeeper software, version 1 (https://www.gene-quantification.de/bestkeeper.html) (accessed on 15 October 2025), candidate reference genes with an SD value below 1 indicate consistent expression patterns [33]. During fruit development, *UBQ12* emerged as the most stably expressed reference gene, despite ranking fourth and sixth according to geNorm and NormFinder, respectively. Across various organ types, *UBQ12* likewise exhibited pronounced stability, ranking fourth by geNorm and third by NormFinder (Table 2). Under diverse abiotic stress treatments, *UBC11* was identified as the top-ranked candidate by both geNorm and BestKeeper; however, NormFinder assigned it to the fourth position (Table 3). Notably, the three algorithms yielded markedly divergent stability rankings for several other reference genes that otherwise displayed high stability under abiotic stress conditions (Supplementary Tables S2–S4).

RefFinder, accessible via the following online source: (http://blooge.cn/RefFinder/) (accessed on 15 October 2025), assesses reference genes based on diverse experimental datasets. This tool effectively combines the NormFinder, geNorm, and BestKeeper methodologies to provide a comprehensive assessment of candidate reference genes [32]. Across various sample types, *PP2A15* and *ARF* were identified as the most reliable reference genes for leaves, while *UBC2* and *UBC11* demonstrated strong stability in stems. In the context of root samples, *RPL6* and *PP2A15* emerged as the most reliable reference genes, while in fruit samples, *RPL21* and *RPL6* demonstrated their superiority as the top-performing reference genes. Notably, when considering the assessment of overall organ stability, *RPL6* and *UBC11* demonstrated remarkable stability (Table 2). The optimal reference genes varied considerably across abiotic stress conditions. Under salt stress, *UBC11* and *UBC2* were identified as the most stable candidates, whereas under alkaline stress, *UBC2* and *GPN1* ranked highest. For drought stress, *UBC2* and *RPL6* emerged as the most suitable reference genes (Supplementary Table S3). Notably, *UBC2* and *UBQ12* consistently ranked among the most stable genes across all three abiotic stress treatments (Table 3).

#### 3.3.3. The Optimal Number of Reference Genes for Normalization in Different Tissues and Under Various Abiotic Conditions

**Figure 6:**
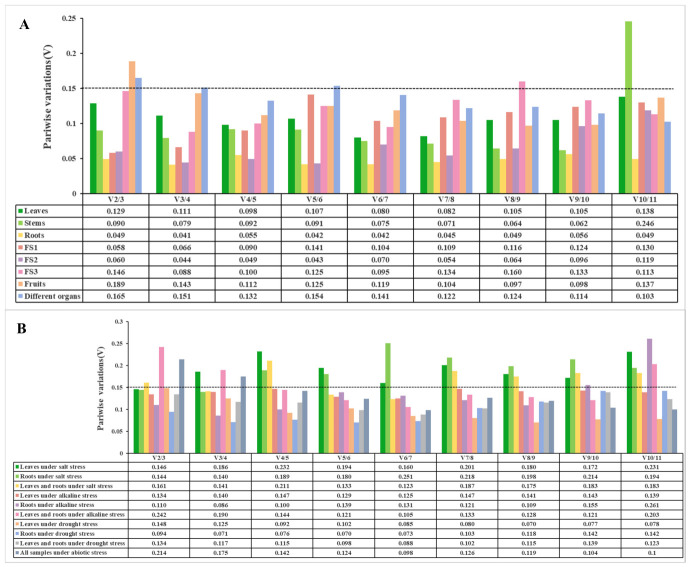
Pairwise variation analyses of the eleven candidate reference genes in different tissues (**A**) and abiotic stress treatments (**B**) of *S. chinensis*. The dotted line means the pairwise variation value is 0.15 (V = 0.15).

To achieve more accurate quantification of target gene expression, the combined use of multiple reference genes for normalization is recommended. To achieve more accurate quantification of target gene expression, the combined use of multiple reference genes for normalization is recommended. The geNorm software evaluates the optimal number of reference genes by calculating pairwise variation values (V*n*/*n* + 1), with values falling below the 0.15 threshold indicating that the inclusion of an additional reference gene provides no meaningful improvement. As shown in Figure 6A, geNorm analysis of fruit developmental stages revealed that three reference genes were required for reliable normalization, with V3/4 = 0.143 falling just below the cutoff. Across different tissue types, four reference genes were necessary, as indicated by V4/5 = 0.132; notably, the V2/3 values for individual tissues already fell below the 0.15 threshold. Under abiotic stress treatments (Figure 6B), the requirements varied by stress type. Leaves and roots subjected to salt stress required a minimum of three reference genes for accurate normalization (V3/4 = 0.141), whereas those exposed to alkaline stress required at least four (V4/5 = 0.144). In contrast, under drought stress, the V2/3 values for both leaves and roots remained below the 0.15 threshold, indicating that two reference genes were sufficient for RT-qPCR data normalization. Overall, accurate normalization of gene expression in leaves and roots across all three abiotic stress conditions required at least four reference genes (V4/5 = 0.142).

### 3.4. Reference Genes Stability Verification

**Figure 7:**
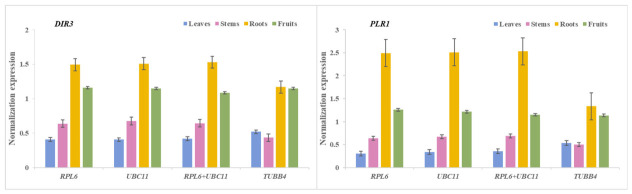
Relative expression of the selected reference genes in different tissues of *S. chinensis*. *DIR3* and *PLR1* were normalized by two sets of the most stable reference genes (*RPL6* and *UBC11*) and the most unstable reference gene (*TUBB4*) in leaves, stems, roots and fruits.

**Figure 8:**
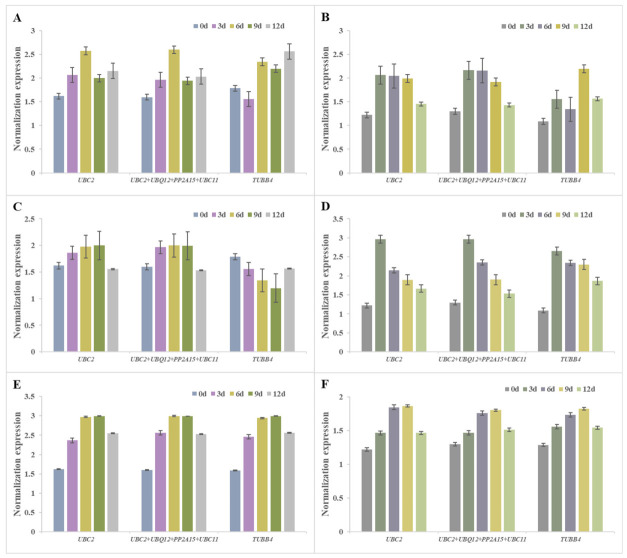
Relative expression of the selected reference gene under different abiotic stresses in *S. chinensis* leaves and roots. *MPK4* was normalized by two sets of the most stable reference genes (*UBC2*, *UBQ12*, *PP2A15* and *UBC11*) and the most unstable reference gene (*TUBB4*) following the three stress treatments after 0, 3, 6, 9, 12 d. (**A**): Leaves under salt stress; (**B**): roots under salt stress; (**C**): leaves under alkaline stress; (**D**): roots under alkaline stress; (**E**): leaves under drought stress; (**F**): roots under drought stress.

To validate the reliability of the selected reference genes, the expression patterns of three target genes (*DIR3*, *PLR1*, and *MPK4*) were examined via RT-qPCR across different tissue types and in both leaves and roots under three abiotic stress conditions. The transcript levels of *DIR3* and *PLR1* were compared across various tissues (Figure 7). When *RPL6*, *UBC11*, or the combination of *RPL6* + *UBC11* were used as reference genes, the expression profiles remained consistent with one another, whereas normalization with *TUBB4* yielded markedly divergent patterns. Under salt and alkaline stress, the expression trends of *MPK4* in leaves and roots diverged substantially depending on whether normalization was performed using the optimal reference gene combination or the least stable reference gene (Figure 8). In contrast, under drought conditions, the expression profiles of *MPK4* in both leaves and roots remained consistent irrespective of the normalization strategy employed. Collectively, these results demonstrate that the use of inappropriate reference genes can introduce substantial bias into target gene expression measurements, thereby compromising experimental accuracy. These findings further validate the selection of the most stable reference genes tailored to distinct tissue types and abiotic stress conditions in *S. chinensis*.

## 4. Discussion

Gene expression analysis has become increasingly central to contemporary molecular biology, playing a pivotal role in deciphering how plants respond to diverse endogenous and environmental signals [34]. The widespread adoption of reverse transcription quantitative PCR (RT-qPCR) has made the stability of internal reference genes a critical determinant of quantitative accuracy. Selecting appropriate reference genes is therefore essential to minimize experimental error in RT-qPCR assays. Currently, four algorithms are widely recognized and routinely employed for evaluating reference gene expression stability: geNorm [29], NormFinder [30], BestKeeper [31], and RefFinder [32]. While geNorm uses pairwise comparison and calculates M values to determine the most stable gene pair, making it suitable for identifying the optimal number of reference genes; NormFinder employs an ANOVA-based model considering both intra- and inter-group variations; BestKeeper relies on raw Ct values and Pearson correlation coefficients, which makes it more sensitive to expression fluctuations; and RefFinder provides a comprehensive ranking by calculating the geometric mean of the three individual rankings. For this experiment, eleven reference genes were chosen to assess their stability across different tissues and under abiotic stress conditions in *S. chinensis*. The stability analysis was conducted using NormFinder, geNorm, RefFinder, and BestKeeper software.

A number of suitable reference genes for RT-qPCR analysis have been identified across diverse experimental conditions, tissue types, and developmental stages in previous studies [5,6,7,22]. For example, Mascia et al. examined the expression stability of reference genes in tomato leaves and roots, identifying *AcTIN* and *UMPK* as genes with consistent expression across these tissue types [14]. In apple, Zhou et al. evaluated the expression stability of five commonly used reference genes across different genotypes, tissues, and fruit developmental stages [21]. Among these, *UBQ* exhibited the most stable expression across all samples tested, establishing it as a preferred reference gene for gene expression analysis in apple. In *Populus tomentosa AcTIN* as the most stable reference gene in stem segments undergoing primary and secondary growth [6]. However, its stability was compromised when assessed across broader developmental stages in *Populus* [35]. These findings underscore that the most suitable reference genes vary considerably across species, tissues, and developmental stages. Consequently, systematic stability evaluation should constitute a prerequisite step in RT-qPCR experimental design. The results of the present study further demonstrate that the optimal reference gene combinations diverge across tissues and experimental conditions, as summarized in Table 2 and Table 3.

The stability rankings of reference genes across different tissues in this study parallel those reported in grape, another vine species. *UBQ12* exhibited consistently stable expression in the present investigation, consistent with that *UBQ* displays stable expression across various grape tissues, developmental stages, and treatment conditions [19]. Similarly, *UBQ* ranked as the second most stable among six candidate reference genes evaluated in leaves and skins of diverse grape varieties [20]. However, the expression stability of *UBQ12* varies considerably across tissue types and developmental stages in walnut [36], suggesting that stable expression of this gene family is not universal among woody perennials. Whether the observed similarity in reference gene stability between S. chinensis and grape reflects their shared vining growth habit remains to be investigated.

In this study, some conventional reference genes, particularly *TUBBA2* and *TUBB4*, did not exhibit high expression stability in *S. chinensis*. Across different tissues, *TUBBA2* ranked only fifth, whereas *TUBB4* showed the lowest stability. This tissue-dependent instability may be related to the biological characteristics of *S. chinensis* as a perennial woody vine. Tubulin genes are not merely constitutively expressed structural genes; they are also involved in cytoskeleton organization, cell division, secondary cell wall formation, and lignification. These processes differ substantially among leaves, stems, roots, and fruits, which may lead to variable expression patterns of *TUBBA2* and *TUBB4* across tissues. In addition, abiotic stresses, especially saline–alkaline stress, can induce cytoskeletal reorganization and thereby alter tubulin gene expression, further reducing their suitability as stable internal controls under stress conditions. Similar observations have been reported in several woody plant species, such as apple [21], in which traditional housekeeping genes, including *AcTIN* and *TUBULIN*, were not always ranked among the most stable reference genes. These results indicate that the stability of classical housekeeping genes is not universal across plant taxa or experimental conditions, particularly in woody perennial species. Therefore, reference genes should be systematically validated for each species and treatment condition rather than selected solely based on their conventional use.

On the other hand, novel identified reference genes were also included such as *RPL* (which encodes proteins with homologous domains) and *UBC* (ubiquitin-conjugating enzyme) in the selection of potential reference genes for this investigation. Based on the experimental findings, it was determined that *RPL6* and *UBC11* showed consistent expression across various organ collections of *S. chinensis*. Moreover, *UBC2* and *UBQ12* demonstrated superior expression stability in collections exposed to abiotic stress. These results indicate that the performance of some new reference genes are superior to traditional reference genes. A similar trend has been previously reported in other studies. For instance, seven novel reference genes in wheat, including Methionine aminopeptidase 1 (*MetAP1*), displayed better expression stability compared to traditional ones such as *AcTIN* and *GAPDH*, especially in different tissues [5]. In *Gossypium hirsutum*, observed that Transmembrane 9 superfamily member 5 (*TMN5*) and Protein trichome birefringence-like 6 (*TBL6*) exhibited more stable expression compared to the traditional genes *PP2A1* and *UBQ14* [23]. In various tissues of *Abelmoschus esculentus* L., *HIS6*, EF-1α, and *UBC5* emerged as the most suitable reference genes [22]. Chen et al. evaluated a panel of reference genes across various tissues of kiwifruit, recommending *UBE2V* as the most suitable reference gene for root, stem, and multi-organ samples [7]. Furthermore, *PP2A* has been shown to exhibit stable expression under diverse abiotic stress conditions and has emerged as a novel reference gene in several plant species. In *Populus euphratica*, *PP2A* maintained stability under drought stress [15] and across a range of abiotic stresses [16]. It was also ranked as the most stable reference gene in *Pennisetum glaucum* under hormone treatments and abiotic stress [17] and in *Hibiscus cannabinus* [37]. Additionally, *PP2A* was identified as the preferred reference gene for *Momordica charantia* under salt stress [18]. Collectively, these studies not only introduced novel reference genes for their respective experimental systems but also identified optimal reference genes tailored to specific stress conditions, providing a valuable resource for future gene expression studies.

The present study provides several insights with broader implications. First, the validated reference genes establish a critical foundation for future RT-qPCR-based functional genomic studies in *S. chinensis*, particularly for lignan biosynthesis genes (e.g., *DIR3*, *PLR1*) and stress-responsive pathways (e.g., *MPK4*), which have long been hindered by the absence of validated internal controls. Second, the differential stability patterns observed may be explained by the distinct biological functions of these genes. The ribosomal protein genes (*RPL6, RPL15, RPL21*) exhibited consistently high stability, likely reflecting their housekeeping role in protein synthesis, which is a fundamental process largely invariant across tissues and stress conditions. Conversely, ubiquitin–proteasome pathway genes (*UBC2, UBC11, UBQ12*) showed condition-dependent variability, consistent with the known differential regulation of protein degradation in response to abiotic stress [27]. Similarly, the β-tubulin genes *TUBB4* and *TUBBA2* ranked among the least stable candidates, likely because cytoskeletal dynamics are highly responsive to developmental and environmental cues, as also reported in other plant species [15,16]. Third, the optimal two-gene combinations (*PP2A15* + *UBC2* for leaves; *RPL6* + *UBC11* for the full tissue panel) yielded V values below 0.15, meeting the widely accepted threshold for reliable normalization [29]. These findings reinforce that reference gene selection must be tailored to specific experimental contexts, and contribute to the growing consensus on the necessity of systematic reference gene validation in non-model plant species.

## 5. Conclusions

Eleven candidate reference genes were evaluated for their expression stability across different tissue types and under abiotic stress conditions in *S. chinensis*. For leaves, the combination of *PP2A15* and *UBC2* was identified as the most stably expressed pair, whereas *UBC2* and *UBC11* proved optimal for stems. *RPL6* and *PP2A15* exhibited the highest stability in roots, and *RPL21* and *RPL6* were the most stable reference genes in fruits. Under salt stress, leaves and roots displayed the greatest stability when *UBC11* and *UBC2* were used in combination. Under alkaline stress, the pairing of *UBC2* and *GPN1* yielded the best normalization results. For leaves and roots subjected to drought stress, the combination of *UBC2* and *RPL6* was the most effective. Across all tissue types in *S. chinensis*, *RPL6* and *UBC11* consistently emerged as the top-performing reference gene pair. Under abiotic stress conditions, *UBC2* and *UBQ12* were identified as the optimal combination. This study represents the first systematic identification of suitable reference genes for *S. chinensis* using RT-qPCR, establishing an essential foundation for future functional gene studies in this species.

## Figures and Tables

**Table 1:** Candidate reference genes, primers and amplification characteristics.

Gene	Gene ID	Primer Sequences (5′→3′)	Amplicon Length (bp)	TM (°C)	*R* ^2^	*E* (%)
*GPN1*	TRINITY_DN24960_c0_g2	5′ATCTCAGAGCCTTTTCCAGTA3′	105	84.2	0.997	99.7
		5′ATGTTCTCAGGGTGATGTAGC3′				
*PP2A15*	TRINITY_DN27677_c1_g1	5′TACTCCTCCCGAGATATGTAAC3′	104	84.4	0.993	102.7
		5′TGTCCTTCTTGGGTAGGTTTT3′				
*RPL6*	TRINITY_DN25507_c0_g5	5′ACTCAGGGCAAGCATTACACC3′	105	83.1	0.999	105.1
		5′AGAAGCAAACCAGAGGCAAGT3′				
*RPL15*	TRINITY_DN31532_c0_g1	5′GCTGCTCCACTCTTCACCATT3′	183	85.7	0.995	105.4
		5′TTGTCCAGACGAAACCGCTCA3′				
*RPL21*	TRINITY_DN28916_c1_g2	5′ATGGCATCTTCCACCCTCGTT3′	185	88.2	0.992	100.9
		5′ATGGCTGCTCCTGTTCTTCG3′				
*TUBBA2*	TRINITY_DN25006_c0_g4	5′TTACACCATTGGCAAGGAGAT3′	102	84.4	0.996	98.6
		5′ACAGCGTTGAATACAAGGAAT3′				
*TUBB4*	TRINITY_DN32951_c2_g5	5′TCGGTATTGGATGTTGTGAGA3′	122	83.9	0.996	102.7
		5′ACTTTGGAAATCAGAAGGGTT3′				
*UBC2*	TRINITY_DN32365_c0_g4	5′ATGTCGCAGCCATACTCACTT3′	128	87.2	0.995	101.6
		5′CTCCCGCACTCTTCTGTTGTA3′				
*UBC11*	TRINITY_DN21226_c0_g1	5′AAGTACGAATCCACGGCAAGG3′	166	84.1	0.995	104.3
		5′TAGCCAGCAGGAGTCCATAGC3′				
*UBQ12*	TRINITY_DN26140_c0_g3	5′AAGTTTCAGAGGAATCCCACC3′	119	82.3	0.994	103.7
		5′AGTTATTGTCTTGCCAGTGAG3′				
*AcTIN*	TRINITY_DN23647_c0_g4	5′GAAGCACTTTCGGTGGACAA3′	185	90.9	0.997	101.6
		5′GGGGTGCTATACTAGCCAAA3′				

**Table 2:** Stability analysis of candidate reference genes in different tissues of *S. chinensis* by NormFinder, BestKeeper and RefFinder.

Group	Rank	NormFinder	M	BestKeeper	SD	CV	RefFinder	Stability
Leaves	1	*PP2A15*	0.032	*RPL6*	0.31	1.45	*PP2A15*	1.57
	2	*UBC2*	0.032	*UBC11*	0.36	1.66	*UBC2*	2.30
	3	*RPL21*	0.235	*UBQ12*	0.50	2.30	*UBQ12*	3.22
	4	*UBQ12*	0.333	*AcTIN*	0.57	2.32	*UBC11*	3.56
	5	*UBC11*	0.348	*TUBB4*	0.64	2.99	*RPL6*	3.81
	6	*RPL15*	0.488	*PP2A15*	0.70	2.73	*RPL21*	5.38
	7	*RPL6*	0.513	*UBC2*	0.73	3.31	*TUBB4*	6.40
	8	*TUBB4*	0.581	*RPL21*	0.75	3.54	*RPL15*	7.67
	9	*TUBBA2*	0.936	*RPL15*	1.02	4.97	*AcTIN*	7.95
	10	*AcTIN*	0.939	*TUBBA2*	1.05	4.45	*TUBBA2*	9.24
	11	*GPN1*	1.356	*GPN1*	1.29	5.22	*GPN1*	11.00
Stems	1	*UBC2*	0.044	*UBC11*	0.32	1.48	*UBC2*	2.21
	2	*UBQ12*	0.044	*TUBB4*	0.36	1.82	*UBC11*	2.21
	3	*UBC11*	0.071	*RPL6*	0.39	1.86	*RPL6*	2.45
	4	*RPL6*	0.078	*AcTIN*	0.42	1.68	*UBQ12*	2.89
	5	*TUBB4*	0.294	*RPL15*	0.49	2.3	*TUBB4*	3.50
	6	*PP2A15*	0.474	*UBC2*	0.59	2.69	*RPL15*	6.44
	7	*RPL15*	0.708	*UBQ12*	0.60	2.81	*PP2A15*	6.45
	8	*RPL21*	0.935	*PP2A15*	0.61	2.49	*AcTIN*	7.75
	9	*TUBBA2*	0.974	*TUBBA2*	0.73	3.08	*RPL21*	8.46
	10	*AcTIN*	1.097	*RPL21*	0.73	3.32	*TUBBA2*	9.24
	11	*GPN1*	2.093	*GPN1*	1.87	7.41	*GPN1*	11.00
Roots	1	*RPL6*	0.023	*GPN1*	0.16	0.67	*RPL6*	2.00
	2	*PP2A15*	0.023	*RPL15*	0.23	1.03	*PP2A15*	2.45
	3	*RPL15*	0.137	*PP2A15*	0.32	1.32	*RPL15*	3.08
	4	*UBC2*	0.192	*RPL6*	0.34	1.63	*AcTIN*	3.83
	5	*TUBBA2*	0.211	*TUBB4*	0.36	1.72	*TUBB4*	3.96
	6	*AcTIN*	0.255	*AcTIN*	0.36	1.53	*UBC2*	5.42
	7	*TUBB4*	0.260	*UBQ12*	0.37	1.76	*GPN1*	5.62
	8	*UBC11*	0.396	*TUBBA2*	0.38	1.55	*TUBBA2*	6.12
	9	*UBQ12*	0.428	*UBC2*	0.41	1.94	*UBQ12*	8.45
	10	*GPN1*	0.478	*UBC11*	0.59	2.74	*UBC11*	8.46
	11	*RPL21*	0.57	*RPL21*	0.61	2.66	*RPL21*	11.00
Fruits	1	*RPL21*	0.302	*UBQ12*	0.39	1.84	*RPL21*	1.32
	2	*TUBBA2*	0.372	*AcTIN*	0.63	2.66	*RPL6*	2.45
	3	*RPL6*	0.417	*RPL21*	0.67	2.74	*UBQ12*	3.22
	4	*UBC11*	0.532	*RPL6*	0.74	3.46	*TUBBA2*	3.25
	5	*AcTIN*	0.635	*UBC11*	0.86	3.95	*AcTIN*	4.16
	6	*UBQ12*	0.655	*UBC2*	0.88	3.97	*UBC11*	4.47
	7	*GPN1*	0.738	*TUBBA2*	0.95	3.92	*GPN1*	7.45
	8	*RPL15*	0.852	*RPL15*	1.00	4.46	*RPL15*	8.00
	9	*UBC2*	0.888	*GPN1*	1.11	4.59	*UBC2*	8.13
	10	*TUBB4*	2.048	*TUBB4*	1.74	7.81	*TUBB4*	10.00
	11	*PP2A15*	2.103	*PP2A15*	1.87	7.77	*PP2A15*	11.00
Different tissues	1	*UBC11*	0.502	*UBQ12*	0.50	2.34	*RPL6*	1.68
	2	*RPL6*	0.540	*RPL6*	0.54	2.52	*UBC11*	1.73
	3	*UBQ12*	0.576	*UBC11*	0.54	2.22	*UBQ12*	2.45
	4	*TUBBA2*	0.627	*AcTIN*	0.69	2.73	*UBC2*	3.31
	5	*UBC2*	0.710	*TUBBA2*	0.75	3.41	*TUBBA2*	4.73
	6	*RPL15*	0.923	*UBC2*	0.81	3.70	*RPL15*	6.24
	7	*GPN1*	1.220	*RPL15*	0.87	4.14	*AcTIN*	6.73
	8	*AcTIN*	1.296	*GPN1*	0.97	4.02	*GPN1*	7.24
	9	*RPL21*	1.378	*PP2A15*	1.02	4.63	*RPL21*	9.24
	10	*PP2A15*	1.737	*RPL21*	1.31	5.30	*PP2A15*	9.74
	11	*TUBB4*	1.847	*TUBB4*	1.36	5.91	*TUBB4*	11.00

**Table 3:** Stability analysis of candidate reference genes for three abiotic stress sets in *S. chinensis* leaves and roots by NormFinder, BestKeeper and RefFinder.

Rank	NormFinder	M	BestKeeper	SD	CV	RefFinder	Stability
1	*UBC2*	0.664	*UBC11*	0.69	2.87	*UBC2*	1.19
2	*UBQ12*	0.810	*UBC2*	0.87	3.61	*UBQ12*	1.86
3	*PP2A15*	0.849	*AcTIN*	0.96	4.07	*UBC11*	3.60
4	*UBC11*	0.948	*UBQ12*	0.96	4.17	*PP2A15*	4.36
5	*TUBB4*	0.984	*PP2A15*	1.09	4.20	*TUBBA2*	5.48
6	*TUBBA2*	1.013	*RPL6*	1.20	5.10	*TUBB4*	5.96
7	*GPN1*	1.036	*TUBB4*	1.24	5.45	*GPN1*	7.05
8	*RPL21*	1.049	*RPL15*	1.38	5.91	*RPL21*	7.09
9	*RPL15*	1.113	*RPL21*	1.45	5.89	*RPL15*	7.90
10	*RPL6*	1.217	*TUBBA2*	1.50	6.04	*AcTIN*	8.54
11	*AcTIN*	1.439	*GPN1*	1.60	6.40	*RPL6*	8.80

## Data Availability

Data available within the article or its Supplementary Materials.

## Supplementary Materials

The following supporting information can be downloaded at: https://www.mdpi.com/article/10.3390/plants15131946/s1, Figure S1: The fruitlets (FS1), fruits at the color transition stage (FS2) and ripe fruits (FS3) of *S. chinensis*; Figure S2: Standard curves of eleven candidate reference genes; Table S1: Stability analysis of candidate reference genes during fruits development of *S. chinensis* by NormFinder, BestKeeper and RefFinder; Table S2: Stability analysis of candidate reference genes under salt stress of *S. chinensis* by NormFinder, BestKeeper and RefFinder; Table S3: Stability analysis of candidate reference genes under alkaline stress of *S. chinensis* by NormFinder, BestKeeper and RefFinder. Table S4: Stability analysis of candidate reference genes under drought stress of *S. chinensis* by NormFinder, BestKeeper and RefFinder.
