# Supplementary material for: Identification and Validation of Reference Genes for Reliable RT-qPCR Normalization in Schisandra chinensis Across Different Tissues and Abiotic Stress Conditions
Source: Plants (Basel). 2026 Jun 24;15(13):1946. doi: 10.3390/plants15131946 (PMC13364142; doi:10.3390/plants15131946)
Supplement: Supplementary file 1 [file plants-15-01946-s001.zip › plants-4304685-supplementary.pdf]

Table S1 Stability analysis of candidate reference genes during fruits development of *S. chinensis* by NormFinder, Bestkeeper and RefFinder

Group	Rank	NormFinder	M	Bestkeeper	SD	CV	RefFinder	Stability
FS1	1	*RPL15*	0.189	*RPL6*	0.31	1.45	*PP2A15*	1.57
	2	*GPN1*	0.258	*UBC11*	0.36	1.66	*UBC2*	2.3
	3	*PP2A15*	0.271	*UBQ12*	0.5	2.3	*UBQ12*	3.22
	4	*AcTIN*	0.348	*AcTIN*	0.57	2.32	*UBC11*	3.56
	5	*RPL21*	0.426	*TUBB4*	0.64	2.99	*RPL6*	3.81
	6	*UBC2*	0.481	*PP2A15*	0.7	2.73	*RPL21*	5.38
	7	*UBQ12*	0.572	*UBC2*	0.73	3.31	*TUBB4*	6.4
	8	*UBC11*	0.697	*RPL21*	0.75	3.54	*RPL15*	7.67
	9	*TUBBA2*	0.739	*RPL15*	1.02	4.97	*AcTIN*	7.95
	10	*TUBB4*	1.108	*TUBBA2*	1.05	4.45	*TUBBA2*	9.24
	11	*RPL6*	1.537	*GPN1*	1.29	5.22	*GPN1*	11
FS2	1	*RPL15*	0.136	*UBQ12*	0.16	0.75	*RPL6*	1.86
	2	*RPL6*	0.196	*TUBB4*	0.36	1.74	*RPL15*	2.66
	3	*RPL21*	0.246	*AcTIN*	0.4	1.63	*RPL21*	3.03
	4	*TUBBA2*	0.281	*PP2A15*	0.57	2.18	*TUBBA2*	4.12
	5	*TUBB4*	0.372	*RPL15*	0.67	2.96	*TUBB4*	4.36
	6	*UBC2*	0.438	*RPL6*	0.8	3.76	*UBC2*	5.73
	7	*PP2A15*	0.521	*RPL21*	0.8	3.32	*UBQ12*	6.04
	8	*GPN1*	0.805	*TUBBA2*	0.86	3.49	*PP2A15*	6.29
	9	*AcTIN*	1.042	*UBC2*	0.96	4.37	*AcTIN*	7.21
	10	*UBC11*	1.053	*GPN1*	1.14	4.55	*GPN1*	8.18
	11	*UBQ12*	1.215	*UBC11*	1.27	5.78	*UBC11*	9.72
FS3	1	*UBC2*	0.133	*PP2A15*	0.47	1.84	*UBC11*	2.82
	2	*RPL6*	0.187	*UBQ12*	0.67	3.06	*RPL15*	2.83
	3	*UBC11*	0.214	*AcTIN*	0.78	3.2	*RPL6*	2.99
	4	*RPL15*	0.35	*RPL15*	0.98	4.16	*UBC2*	3.31
	5	*RPL21*	0.433	*RPL6*	1.03	4.72	*RPL21*	3.5
	6	*UBQ12*	0.536	*RPL21*	1.07	4.23	*PP2A15*	4.45
	7	*PP2A15*	0.813	*UBC11*	1.23	5.55	*UBQ12*	4.56
	8	*TUBBA2*	0.946	*UBC2*	1.32	5.97	*AcTIN*	7.95
	9	*TUBB4*	1.275	*TUBBA2*	1.75	7.02	*TUBBA2*	7.97
	10	*GPN1*	1.318	*TUBB4*	1.92	8.84	*TUBB4*	9.24
	11	*AcTIN*	2.409	*GPN1*	1.95	7.96	*GPN1*	10.24
Fruits	1	*RPL21*	0.302	*UBQ12*	0.39	1.84	*RPL21*	1.32
	2	*TUBBA2*	0.372	*AcTIN*	0.63	2.66	*RPL6*	2.45
	3	*RPL6*	0.417	*RPL21*	0.67	2.74	*UBQ12*	3.22
	4	*UBC11*	0.532	*RPL6*	0.74	3.46	*TUBBA2*	3.25
	5	*AcTIN*	0.635	*UBC11*	0.86	3.95	*AcTIN*	4.16
	6	*UBQ12*	0.655	*UBC2*	0.88	3.97	*UBC11*	4.47
	7	*GPN1*	0.738	*TUBBA2*	0.95	3.92	*GPN1*	7.45
	8	*RPL15*	0.852	*RPL15*	1.00	4.46	*RPL15*	8.00
	9	*UBC2*	0.888	*GPN1*	1.11	4.59	*UBC2*	8.13
	10	*TUBB4*	2.048	*TUBB4*	1.74	7.81	*TUBB4*	10.00
	11	*PP2A15*	2.103	*PP2A15*	1.87	7.77	*PP2A15*	11.00

Table S2 Stability analysis of candidate reference genes under salt stress of *S. chinensis* by NormFinder, Bestkeeper and RefFinder

Group	Rank	NormFinder	M	Bestkeeper	SD	CV	RefFinder	Stability
Leaves	1	*TUBBA2*	0.231	*UBQ12*	0.46	2.01	*TUBBA2*	2.11
	2	*UBC11*	0.399	*UBC11*	0.50	2.08	*UBC11*	2.21
	3	*UBC2*	0.501	*UBC2*	0.52	2.16	*UBC2*	2.28
	4	*GPN1*	0.512	*AcTIN*	0.70	2.92	*UBQ12*	2.34
	5	*TUBB4*	0.616	*PP2A15*	0.85	3.24	*GPN1*	5.18
	6	*UBQ12*	0.669	*TUBBA2*	0.81	3.35	*TUBB4*	5.96
	7	*PP2A15*	0.670	*TUBB4*	1.10	4.79	*PP2A15*	6.74
	8	*AcTIN*	1.042	*RPL21*	1.11	4.60	*AcTIN*	7.14
	9	*RPL21*	1.115	*GPN1*	1.13	4.69	*RPL21*	8.24
	10	*RPL15*	1.297	*RPL15*	1.33	5.70	*RPL15*	10.00
	11	*RPL6*	1.744	*RPL6*	1.40	5.77	*RPL6*	11.00
Roots	1	*RPL6*	0.390	*AcTIN*	0.33	1.38	*RPL6*	1.68
	2	*UBC2*	0.583	*RPL6*	1.09	4.63	*UBC2*	2.45
	3	*UBC11*	0.636	*UBC2*	1.11	4.53	*UBC11*	2.59
	4	*UBQ12*	0.682	*RPL21*	1.22	4.85	*UBQ12*	3.25
	5	*PP2A15*	0.728	*UBC11*	1.26	5.12	*AcTIN*	5.05
	6	*RPL21*	0.937	*RPL15*	1.26	5.28	*PP2A15*	5.62
	7	*TUBB4*	0.967	*UBQ12*	1.35	5.76	*RPL21*	6.24
	8	*RPL15*	0.997	*PP2A15*	1.40	5.38	*TUBB4*	6.90
	9	*AcTIN*	1.122	*TUBB4*	1.49	6.50	*RPL15*	7.20
	10	*TUBBA2*	1.321	*TUBBA2*	1.53	6.15	*TUBBA2*	10.00
	11	*GPN1*	1.451	*GPN1*	2.01	7.96	*GPN1*	11.00
Leaves and Roots	1	*UBC11*	0.505	*AcTIN*	0.55	2.33	*UBC11*	1.32
	2	*UBC2*	0.520	*UBC2*	0.81	3.35	*UBC2*	2.21
	3	*UBQ12*	0.672	*UBC11*	0.86	3.56	*UBQ12*	2.45
	4	*PP2A15*	0.690	*UBQ12*	0.90	3.91	*PP2A15*	4.23
	5	*TUBB4*	0.827	*PP2A15*	1.13	4.31	*AcTIN*	5.32
	6	*TUBBA2*	0.895	*TUBBA2*	1.24	5.06	*TUBB4*	5.79
	7	*RPL21*	1.032	*RPL6*	1.27	5.29	*TUBBA2*	6.00
	8	*AcTIN*	1.062	*RPL21*	1.28	5.20	*RPL21*	7.48
	9	*GPN1*	1.085	*TUBB4*	1.29	5.64	*GPN1*	8.63
	10	*RPL15*	1.089	*RPL15*	1.35	5.71	*RPL15*	9.49
	11	*RPL6*	1.335	*GPN1*	1.57	6.36	*RPL6*	9.82

Table S3 Stability analysis of candidate reference genes under alkaline stress of *S. chinensis* by NormFinder, Bestkeeper and RefFinder

Group	Rank	NormFinder	M	Bestkeeper	SD	CV	RefFinder	Stability
Leaves	1	*TUBBA2*	0.267	*UBQ12*	0.49	2.22	*RPL21*	2.21
	2	*RPL21*	0.419	*RPL15*	0.66	3.03	*UBC2*	2.59
	3	*UBC2*	0.435	*UBC11*	0.70	2.98	*UBC11*	2.91
	4	*RPL15*	0.479	*RPL21*	0.75	3.27	*TUBBA2*	3.03
	5	*GPN1*	0.518	*UBC2*	0.83	3.59	*RPL15*	3.56
	6	*UBC11*	0.541	*GPN1*	0.83	3.53	*UBQ12*	4.49
	7	*TUBB4*	0.660	*TUBBA2*	0.85	3.63	*GPN1*	5.96
	8	*PP2A15*	0.813	*TUBB4*	1.07	4.76	*TUBB4*	7.48
	9	*UBQ12*	0.986	*AcTIN*	1.25	5.17	*PP2A15*	8.71
	10	*AcTIN*	1.022	*PP2A15*	1.48	5.74	*AcTIN*	9.74
	11	*RPL6*	1.721	*RPL6*	1.75	7.50	*RPL6*	11.00
Roots	1	*TUBBA2*	0.198	*UBC11*	0.32	1.34	*TUBBA2*	2.34
	2	*GPN1*	0.429	*AcTIN*	0.40	1.64	*GPN1*	3.44
	3	*PP2A15*	0.564	*RPL21*	0.46	1.77	*AcTIN*	3.72
	4	*AcTIN*	0.825	*RPL15*	0.57	2.33	*PP2A15*	4.74
	5	*RPL21*	1.005	*GPN1*	0.72	2.70	*UBC2*	4.86
	6	*RPL6*	1.066	*TUBBA2*	0.98	3.66	*RPL21*	5.10
	7	*TUBB4*	1.395	*PP2A15*	1.01	3.84	*UBQ12*	5.46
	8	*UBC2*	1.416	*RPL6*	1.47	6.14	*UBC11*	5.62
	9	*UBQ12*	1.457	*TUBB4*	1.68	7.31	*RPL6*	5.83
	10	*UBC11*	1.538	*UBC2*	1.71	6.78	*TUBB4*	6.24
	11	*RPL15*	1.648	*UBQ12*	1.73	7.14	*RPL15*	8.54
Leaves and Roots	1	*GPN1*	0.815	*UBC11*	0.48	2.05	*UBC2*	2.06
	2	*PP2A15*	0.956	*AcTIN*	0.83	3.41	*GPN1*	2.38
	3	*UBC2*	0.979	*UBC2*	1.19	4.92	*PP2A15*	3.60
	4	*TUBBA2*	1.098	*PP2A15*	1.20	4.63	*UBQ12*	3.66
	5	*RPL21*	1.123	*UBQ12*	1.22	5.24	*TUBBA2*	5.07
	6	*UBQ12*	1.174	*TUBB4*	1.32	5.80	*UBC11*	5.32
	7	*TUBB4*	1.185	*RPL15*	1.34	5.82	*RPL21*	5.48
	8	*RPL15*	1.272	*GPN1*	1.57	6.25	*AcTIN*	6.18
	9	*AcTIN*	1.283	*RPL21*	1.58	6.49	*RPL15*	7.20
	10	*UBC11*	1.320	*RPL6*	1.61	6.81	*TUBB4*	7.36
	11	*RPL6*	1.468	*TUBBA2*	1.77	7.03	*RPL6*	10.74

Table S4 Stability analysis of candidate reference genes under drought stress of *S. chinensis* by NormFinder, Bestkeeper and RefFinder

Group	Rank	NormFinder	M	Bestkeeper	SD	CV	RefFinder	Stability
Leaves	1	*RPL15*	0.340	*RPL21*	0.61	2.61	*RPL6*	1.86
	2	*RPL6*	0.370	*RPL15*	0.64	2.92	*UBC2*	2.21
	3	*UBC2*	0.413	*UBQ12*	0.82	3.71	*RPL15*	2.74
	4	*TUBBA2*	0.531	*UBC2*	1.02	4.35	*RPL21*	3.50
	5	*RPL21*	0.569	*UBC11*	1.09	4.60	*TUBBA2*	4.53
	6	*UBC11*	0.716	*RPL6*	1.22	5.38	*UBC11*	4.82
	7	*UBQ12*	0.723	*TUBBA2*	1.26	5.40	*UBQ12*	4.92
	8	*GPN1*	0.730	*PP2A15*	1.32	5.23	*TUBB4*	8.92
	9	*TUBB4*	0.844	*AcTIN*	1.35	5.78	*GPN1*	8.97
	10	*PP2A15*	1.439	*GPN1*	1.44	6.06	*PP2A15*	9.46
	11	*AcTIN*	2.141	*TUBB4*	1.59	6.97	*AcTIN*	10.46
Roots	1	*UBC2*	0.229	*RPL6*	0.16	0.68	*UBC2*	1.19
	2	*UBQ12*	0.272	*UBC2*	0.23	0.97	*UBQ12*	1.86
	3	*RPL15*	0.330	*UBQ12*	0.24	1.05	*RPL15*	3.22
	4	*PP2A15*	0.335	*RPL15*	0.29	1.17	*RPL6*	3.44
	5	*RPL21*	0.369	*RPL21*	0.35	1.34	*PP2A15*	5.09
	6	*UBC11*	0.418	*UBC11*	0.39	1.61	*UBC11*	5.73
	7	*RPL6*	0.429	*PP2A15*	0.44	1.66	*RPL21*	5.92
	8	*TUBB4*	0.434	*TUBB4*	0.60	2.62	*TUBB4*	8.00
	9	*TUBBA2*	0.619	*TUBBA2*	0.61	2.32	*TUBBA2*	9.00
	10	*GPN1*	0.860	*GPN1*	0.92	3.47	*GPN1*	10.00
	11	*AcTIN*	1.071	*AcTIN*	0.97	4.22	*AcTIN*	11.00
Leaves and Roots	1	*UBC2*	0.423	*RPL6*	0.75	3.29	*UBC2*	1.19
	2	*UBQ12*	0.492	*UBC2*	0.77	3.23	*RPL6*	1.73
	3	*RPL6*	0.596	*UBC11*	0.77	3.23	*UBQ12*	2.83
	4	*UBC11*	0.785	*UBQ12*	0.86	3.76	*UBC11*	3.46
	5	*RPL15*	0.935	*PP2A15*	0.98	3.81	*RPL15*	5.62
	6	*PP2A15*	0.974	*TUBB4*	1.09	4.77	*PP2A15*	7.02
	7	*TUBBA2*	1.003	*AcTIN*	1.16	5.00	*TUBBA2*	7.17
	8	*TUBB4*	1.008	*RPL15*	1.46	6.21	*TUBB4*	7.44
	9	*RPL21*	1.022	*TUBBA2*	1.49	5.99	*RPL21*	7.84
	10	*GPN1*	1.115	*RPL21*	1.49	6.01	*AcTIN*	9.82
	11	*AcTIN*	1.844	*GPN1*	1.59	6.31	*GPN1*	10.24

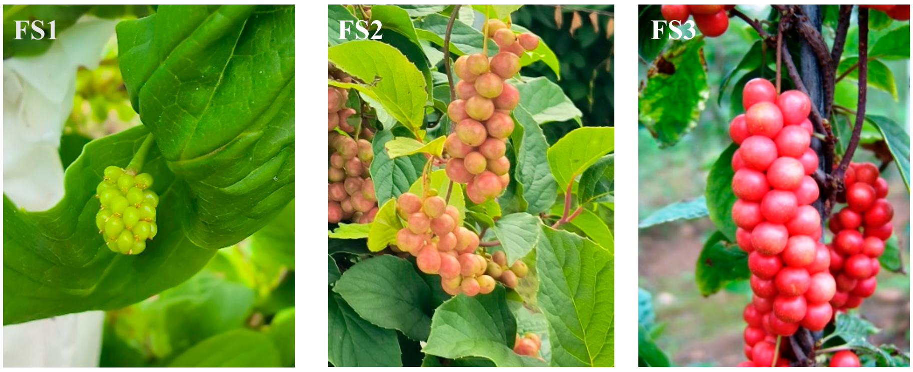

Figure S1 The fruitlets (FS1), fruits at the color transition stage (FS2) and ripe fruits (FS3) of *S. chinensis*

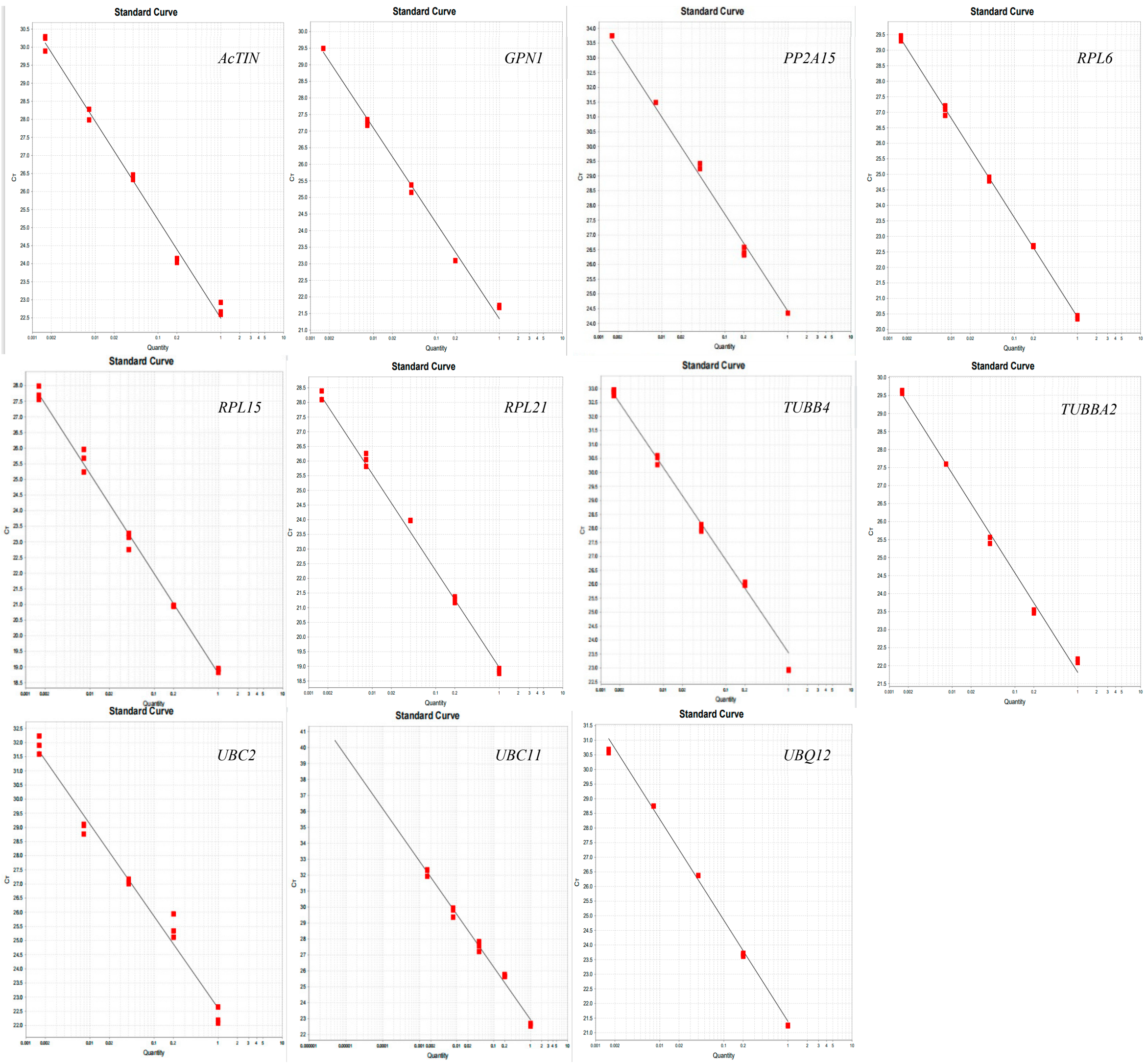

Figure S2 Standard curves of eleven candidate reference genes
